# New Frontiers for Chemical Ecology: Reaffirming a Commitment to the Göteborg Resolution: Guest Editorial: *The Bengaluru Consortium

**DOI:** 10.1007/s10886-016-0813-9

**Published:** 2017-02-09

**Authors:** Shannon B. Olsson, The Bengaluru Consortium

**Affiliations:** National Centre for Biological Sciences, Tata Institute of Fundamental Research, Bangalore, India

In August 1989, members of the International Society for Chemical Ecology (ISCE) assembled in Göteborg, Sweden and unanimously adopted a resolution that still graces the website of the ISCE today. The members expressed a determination to accelerate the search for new compounds from nature, and emphasized the need to focus on the tropics, where biodiversity is highest. In a subsequent guest editorial, Eisner and Meinwald (1990) suggested that a coordinated effort across the world is necessary to build upon the Göteborg Resolution, involving representatives from industry, academia, health, and government from both the developed and developing world.

Today, 27 years later, the ISCE has grown to hundreds of members, and there are thriving regional chemical ecological societies representing Latin America (ALAEQ) and the Asian-Pacific region (APACE), among several others. Yet, despite this progress, there remains a paucity of chemical ecologists from many biodiversity-rich regions around the world. This year, a workshop was held in Bengaluru, India, comprising scientists, students, and industry representatives from 20 Indian states and 5 continents to address this very issue. India not only has a rich diversity of ecosystems, but also a long cultural history of utilizing its diverse chemical patrimony for food, health, and agriculture. During the meeting, participants discussed what is needed to invigorate chemical ecology in understudied regions of the world such as India, and what these regions offer the field and its future. They also discussed how members of the field can increase collaborations and research for the preservation of the world’s biodiversity, and promote the use of semiochemicals in agriculture, medicine, and industry. Specifically, participants hoped to offer a road map to reaffirm and reinvigorate the Göteborg Resolution, from which chemical ecology can be recognized as a global field, embedded within and inspired by the biological diversity of our planet. This perspective provides a summary of outcomes that emerged from the workshop discussions, which also have relevance to many large, rapidly developing and biodiversity-rich countries worldwide, such as Brazil, Kenya, and China.

## Building Regional Capacity

To increase collaboration and build capacity in regions where chemical ecology is not well established, direct interaction is needed between international societies such as the ISCE and regional societies through joint meetings, social media interaction, and travel and training funding opportunities for promising scientists. Such efforts need to be supported by funding agencies within these regions, initiating specific grant programs for young investigators in the field. Financial support needs to be made available to establish national societies, create advanced infrastructures, and facilitate networking. Recent advances in information technology provide opportunities for open access databases featuring human, chemical, biological, and technological resources and developments relevant to the field. While some chemical ecological databases exist, comprehensive international collections for the field are not readily available. These resources would be invaluable to burgeoning chemical ecology programs that might require expert advice and assistance to establish themselves.

## Harmonizing Chemical Ecology with Public Policy

India and other developing countries face important challenges regarding urbanization, environmental change, disease outbreaks, and food security, for which chemical ecological solutions must be both sought and tested. In particular, the field should encourage more strategic links between academia and public stakeholders so that the applied aspects of chemical ecology reflect the vision and direction of society in general. For example, chemical ecologists could collectively, through regional teams or international societies, promote the development of public policy to conserve useful chemical resources and encourage policies for important societal concerns such as pollution detection, industrial effluents, and food residues. Chemical ecologists also should capitalize on hot spots for public concern in biodiverse regions, employing newspapers, social digital media, and the use of local languages where possible. Identification of new semiochemicals in these regions can lead to biotechnological applications in chemical industries, biomedical research, agriculture, forestry, conservation, and many others. Therefore, chemical ecology research also could serve as a driver for the development of semiochemical technologies for a range of applications, in India and beyond.

## Growing Chemical Ecology Through Education

In developing regions, chemical ecology, or more specifically the chemical basis of biological interactions, is largely absent from basic science curricula in the country. In many cases across the world, biodiversity hotspots are regions where university training is scarce, but increasing capacity for chemical ecology in these regions could leverage increased biodiversity research and integrate local traditional and ecological knowledge that in turn would benefit the field.

To do this, chemical ecologists need to generate a curriculum and leverage online media to accelerate learning worldwide, including a tool for recruiting students and chemists. We need more international training courses, with hands-on training and corresponding theory to create greater competence in the field. Chemical ecology studies should also be encouraged at introductory levels of education at which chemistry and ecology are initially taught. Courses should include a focus on the development of taxonomic and natural products knowledge in biodiverse regions, including indigenous knowledge, with outreach programmes for creating public awareness for conservation of natural resources. Given the alarming reduction of natural products chemistry as a viable field, an urgent call to promote the field for chemists is also proposed. The loss of interested and dedicated chemists would be disastrous for the future of chemical ecology, where the bulk of natural products, especially in the tropics, are yet to be discovered.

The Indian workshop stimulated several specific discussions among the participants to increase collaborative efforts in India, which we hope can serve other chemical ecology programs across the world. In the end, the most promising outcome of this exercise was a renewed enthusiasm to share and learn experiences related to chemical ecology, promote this field, drive its progress, and expand its reach. Indeed, this was the goal in 1989, and it is inspiring to note 27 years later that this ethos is still a fundamental aspect of our field as its reach extends across the globe.
